# Studies on the Virucidal Effects of UV-C of 233 nm and 275 nm Wavelengths

**DOI:** 10.3390/v16121904

**Published:** 2024-12-11

**Authors:** Jessica Kohs, Tom Lichtenthäler, Carolyn Gouma, Hyun Kyong Cho, Andreas Reith, Axel Kramer, Sven Reiche, Paula Zwicker

**Affiliations:** 1 Department of Experimental Animal Facilities and Biorisk Management (ATB), Friedrich-Loeffler-Institut, Federal Research Institute for Animal Health, Südufer 10, 17493 Greifswald Insel Riems, Germany; 2 Institute of Hygiene and Environmental Medicine, University Medicine Greifswald, Ferdinand-Sauerbruch-Str., 17475 Greifswald, Germany; 3 Ferdinand-Braun-Institut gGmbH, Leibniz-Institut Für Höchstfrequenztechnik, Gustav-Kirchhoff-Str. 4, 12489 Berlin, Germany; 4 ams OSRAM International GmbH, Leibnizstr. 4, 93055 Regensburg, Germany

**Keywords:** far-UV-C, UV-C disinfection, antiseptics, room disinfection, virucidal activity, LED UV-C emitters, prophylactic measures, viral transmission reduction

## Abstract

Among the physical decontamination methods, treatment with ultraviolet (UV) radiation is a suitable means of preventing viral infections. Mercury vapor lamps (254 nm) used for room decontamination are potentially damaging to human skin (radiation) and harmful to the environment (mercury). Therefore, other UV-C wavelengths (100–280 nm) may be effective for virus inactivation on skin without damaging it, e.g., far-UV-C radiation with a wavelength of 233 nm, which is absorbed in the outer layer of the skin and thus does not reach the deeper layers of the skin. For room disinfection, 275 nm UV-C LED lamps could be a more environmentally friendly alternative, since toxic mercury is avoided. A carrier test using multiple viruses was used to determine the TCID_50_/mL value on stainless steel, PVC, and glass carriers. In addition to the inactivation kinetics (233 nm), the necessary UV-C dose for 4 lg inactivation (275 nm) was investigated. The impact of irradiance on the inactivation efficacy was also assessed. The inactivation of the viruses was a function of the radiation dose. UV-C-radiation at 233 nm (80 mJ/cm^2^) inactivated from 1.49 ± 0.08 to 4.28 ± 0.18 lg depending on the virus used. To achieve a 4 lg inactivation (275 nm) for enveloped viruses, doses of up to 70 mJ/cm^2^ (SuHV-1) were sufficient. For non-enveloped viruses, a maximum dose of 600 mJ/cm^2^ (MS2) was necessary. Enveloped viruses were inactivated with lower doses compared to non-enveloped viruses. Higher radiation doses were required for inactivation at 275 nm in comparison to 254 nm. A more environmentally friendly alternative to mercury vapor lamps is available with 275 nm LED emitters. Radiation at 233 nm could serve as an additional prophylactic or therapeutic measure for virus inactivation in direct contact with human skin.

## 1. Introduction

Viral infections are an inevitable part of life. While infection with some human viruses only causes mild symptoms, others can lead to serious diseases. Recently, SARS-CoV-2 has had a significant impact on global health, social life, and the economy. For this purpose, the prevention and treatment of viral infections are essential, especially for new pathogens but also for known pathogens with newly evolved characteristics. Preventive and therapeutic methods must consider the individual biology of viruses, entry pathways, and infection strategies, as well as transmission. Modes of transmission differ even among respiratory viruses and include direct contact, indirect contact (via fomites), droplets, and aerosols. The relative importance of each mode depends on the virus strain and a multitude of environmental factors that are insufficiently characterized for most viruses. Generally, the most effective prevention strategies should consider all transmission pathways [1,2]. An effective measure against infection with an airborne virus is the removal of virus-carrying aerosols or droplets by means of air filtration, which is usually carried out by HEPA filters in HVAC systems [3] or, to a lesser extent, by wearing face masks [4].

Another physical method is inactivation with ultraviolet (UV) radiation, which has been used for room irradiation for decades [5]. But traditional technologies for UV room irradiation have significant flaws, which have prevented it from being used more widely. For example, traditional UV-C lamps with an emission peak at 254 nm contain the toxic metal mercury. In the EU, the sale of electronic products containing mercury has been prohibited. Furthermore, such products have restricted light designs due to the size of the lamps [6], which, even if they are small, are still larger than LEDs, limiting their use for special applications, e.g., in endoscopes. Most importantly though, the microbicidal and virucidal effect of UV-C radiation is primarily attributed to DNA damage, which can also harm human cells [7]. Common 254 nm lamps and other wavelengths > 254 nm can cause various types of damage in nucleic acids. In humans, the most common type is dimerization of pyrimidines, resulting in the formation of cyclobutene pyrimidine dimers (CPD) and pyrimidine (6-4) pyrimidone photoproducts (6-4PP). Furthermore, DNA damage is not only caused by direct absorption but can also be mediated by photosensitizers, which transfer absorbed energy [8]. Such UV-induced DNA damage can be directly linked to melanoma cancer formation [8,9]. That is why UV-C radiation is only used for air, water, and surface disinfection or in upper rooms without the risk of radiation exposure to humans. One possibility to overcome the negative properties of 254 nm mercury vapor lamps, particularly the toxicity of mercury and the large size of the lamps, could be the use of LEDs for UV-C generation. In the present study, the virucidal efficacy of UV-C LEDs as an alternative to conventionally used 254 nm UV-C for air disinfection was tested. As LEDs with longer wavelengths have a higher optical output than LEDs with a wavelength of 260 nm—the external quantum efficiency (%) rises from about 0.01 to nearly 100 with the increase in the wavelength (220–400 nm)—and as they have a longer lifespan than mercury vapor lamps (up to 25,000 h vs. 1000–12,000 h) with a 254 nm peak wavelength, these studies were performed with LEDs of 275 nm [10,11,12,13]. This wavelength is close to the absorption maximum of proteins at 280 nm [14] but is, as with 254 nm radiation, not safe for the use when persons can come into direct contact with the radiation.

Another possibility of reducing the transmission of viruses is to inactivate them directly at their entry site or their place of origin, e.g., in the mouth/throat of patients, using a skin-compatible wavelength (e.g., 233 nm) generated by LEDs. The upper respiratory tract is a prime entry site for different viruses. Inactivation of viral loads before they enter can prevent a severe infection; this can even have a therapeutic benefit, as seen with gargling with antiseptic mouthwashes or ointments, sprays, and solutions for nose antisepsis [15]. The efficacy of 233 nm far-UV-C radiation against bacteria of different genera or strains, in addition to its higher skin compatibility in comparison to 254 nm radiation, has already been demonstrated [16,17]. Zwicker et al. and Sicher et al. have shown that a dose of 60–80 mJ/cm^2^ of 233 nm radiation is more skin-compatible than the use of 254 nm or UV-B radiation in regard to 6-4PP and CPD damage when irradiating reconstructed human epidermis. Minor damage disappeared within 24 h. Multiple irradiation (daily irradiation for four days) with 80 mJ/cm^2^ did not induce an increase in 6-4PP and CPD. Excised human skin was more sensitive. However, a dose of 60 mJ/cm^2^ caused only minor damage. The differences compared to 254 nm radiation occur because radiation with a 233 nm peak wavelength is highly absorbed by the outer layer of the skin and thus cannot reach the deeper layers of the skin; thus, 233 nm radiation is more tolerable [18]. A dose of from 60 to 80 mJ/cm^2^ was sufficient to inactivate up to 5 lg of various bacteria, for instance, methicillin-sensitive and methicillin-resistant *Staphylococcus aureus*, *Pseudomonas aeruginosa*, and *Klebsiella pneumoniae*, among others. UV-C radiation at 222 nm from excimer lamps was markedly less efficient. Using matrices such as artificial sweat, artificial saliva, or wound exudate reduced the inactivation efficacy of all wavelengths due to absorption processes. However, solutions containing salts as the main ingredients still enable a reduction of >3 lg [16,17]. Unfortunately, the effect of these matrices on DNA damage has not yet been studied.

The aim of the current study was to investigate the inactivation efficacy of UV-C LED radiation with a peak wavelength of 233 nm on various viruses as a potential application in virus infection prevention and as a supportive treatment in special cases, e.g., dental treatments, as a protective measure for treating staff. Since the efficacy and tolerability of UV-C partly depends on the wavelength (due to different interactions with DNA/RNA and proteins), proof of the virucidal effectiveness of 233 nm radiation is required but has not been elucidated until now. The effectiveness of 275 nm radiation against a wide range of viruses was also determined in order to check whether this wavelength is suitable for treating air and surfaces. To achieve this, it is necessary to know the dose required for inactivation. This is why for 275 nm radiation produced by an LED, the necessary dose for 4 lg reduction (according to DIN EN17272 for room disinfection) has to be determined to ensure that this radiation is an adequate alternative.

In contrast to conventionally used mercury vapor (254 nm) or excimer lamps (222 nm), LED spotlights are more flexible in their design, allowing for new areas of application that would not be possible with bulky mercury vapor or excimer lamps.

The efficacy of UV-C virus inactivation by LED lamps of 233 nm and 275 nm radiation was assessed using a carrier test and calculating the TCID_50_/mL value without and after UV-C irradiation using the following viruses: Human adenovirus 5 (HAdV-5), Bovine coronavirus (BCoV), Feline coronavirus (FCoV), Murine norovirus 1 (MNV), Feline calicivirus (FCV), Poliovirus type 1 (PV-1), Emesvirus zinderi (Escherichia phage, MS2), Modified Vaccinia Ankara (MVA), Mammalian orthoreovirus 1 (ORV-1), Severe acute respiratory syndrome coronavirus type 2 (SARS-CoV-2), Suide alphaherpesvirus 1 (SuHV-1), Indiana vesiculovirus (VSIV), Human coronavirus OC43 (HCoV OC43), and Influenza A virus (H1N1). To answer the question as to whether the surface has an effect on inactivation, three carrier materials (stainless steel, PVC, and glass) were used and compared. The different characteristics of the test viruses (genome, envelope, and strain) and the required irradiance for their inactivation were further assessed.

## 2. Materials and Methods

### 2.1. Cell Culture/Virus Propagation

For each virus, the appropriate cell line (Table 1) was established and maintained with media and 10% fetal calf serum (FCS) unless otherwise mentioned (Table 1). The Level of Quantitation (LLQ) was ca. 1.8 log_10_ (lg) for all viruses, except for H1N1 and OC43, with an LLQ of 2.47 lg. The LLQ was determined using the method of TCID_50_ calculation according to Spearman and Kärber and is characterized by the lowest TCID_50_ that can be determined, meaning that only one of the technical replicates treated with the virus suspension is positive for cytopathic effects (CPEs). The initial titer (Table 1) indicates the concentration that was applied to the carriers.

For virus amplification, T75 cm^2^ cell culture flasks (Greiner/Sarstedt, Nürmbrecht, Germany) were seeded with a suitable number of cells and infected with the respective virus the following day when the cell density reached a confluency of ~80%. For this, the supernatant was discarded, the cells were washed with 5 mL medium without (*w*/*o*) FCS or with phosphate-buffered saline containing Ca^2+^/Mg^2+^ (wPBS) and then infected by the addition of 6 mL of the virus dilution (1:20). After an incubation time of 60 min (37 °C, 5% CO_2_, 95% rH), 24 mL of medium containing 2% FCS (or 5% Panexin CD and 1 µg/mL TPCK-treated Trypsin for H1N1) was added. Over the next 1–5 d, virus growth was assessed for a cytopathic effect (CPE). When a CPE of about 75% was reached, the culture was frozen at −80 °C. Afterwards, the cell culture flask was thawed, and the supernatant was centrifuged at 340× *g* for 10 min. For carrier tests with lower FCS concentrations, the cell culture was flushed with PBS once, and PBS was added to the cells before freezing at −80 °C to obtain a virus aliquot with no FCS.

The purified virus suspension was aliquoted and refrozen at −80 °C. To quantify the titer, an aliquot was thawed and titrated in medium (2% FCS) on a 96-well plate with cells seeded the day before. The final titer was determined after 3–7 days, depending on the virus, as the TCID_50_/mL value [19,20].

### 2.2. Emesvirus Zinderi MS2 Multiplication

For propagation of the MS2 phage, 300 µL of *Escherichia (E.) coli* was incubated with 300 µL of MS2 phage suspension in a reaction tube for 10 min at room temperature (RT). Three centrifuge tubes were filled with soft agar and 200 µL of the *E. coli* phage suspension. The tubes were briefly swirled and poured onto three agar plates. After solidification, the plates were incubated overnight in an incubator (37 °C, 95% RH). The following day, the lysed culture was transferred into a 500 mL flask. The addition of 30 mL of SM buffer (NaCl, Mg_2_SO_4_ × 7H_2_O, 1 M Tris HCl) produced a viscous suspension, which became a homogeneous liquid culture after stirring on a magnetic stirrer for 4 h. The liquid mass was centrifuged at 3200× *g* for 30 min. The supernatant was filtered (syringe filter: 0.45 µm) to remove the *E. coli* and stored at 4 °C for a maximum of four weeks.

### 2.3. Carrier Test

**Figure 1:**
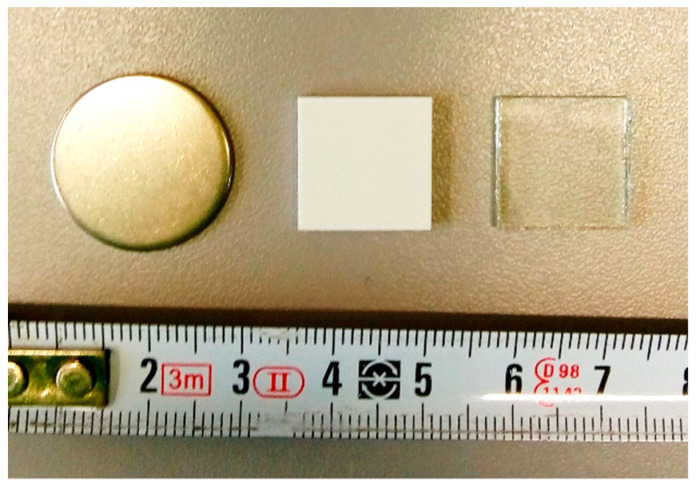
Carriers (stainless steel Ø 20 mm, PVC, and glass 15 × 15 mm).

The carrier test, based on DIN EN regulations and optimized for the application of UV-C radiation [16,17], was performed by spreading 50 µL of virus suspension in cell culture medium with 2% FCS on a defined carrier (stainless steel 1. 4301 according to EN 10088. 1; KömaDur^®^ ES PVC; glass from GK Formblech GmbH, Berlin, Germany, Figure 1) and dried (30 min, room temperature of 22.5 ± 0.64 °C, 37.43 ± 14.40% humidity in an airflow cabinet) before irradiation with UV-C radiation. Drying was checked visually. For testing the impact of protein in the medium, further tests with FCoV in cell culture medium with 0.3% FCS (233 nm and 254 nm) followed, using 233 nm and 254 nm radiation since the protein content plays a role due to absorption, especially at lower wavelengths. Inactivation kinetics were assessed using steel carriers. At each irradiation time, control tests were carried out that were not irradiated but were stored for the same time under the same conditions (temperature, relative humidity) so that the natural inactivation did not influence the calculated values. After the irradiation, the carriers were rinsed in 1 mL of FCS-free medium. If necessary, three samples were pooled. The supernatant was titrated in medium with 2% FCS (or 5% Panexin CD and 1 µg/mL TPCK-treated Trypsin for H1N1) and transferred to a confluent 96-well plate. The microtiter plate was incubated at 37.0 °C, 5% CO_2_, and 95.0% RH under daily microscopic control. After the development of a CPE, the titer was determined. Depending on the virus, the final titer could be determined after 3–7 days and calculated using the TCID_50_/mL value according to Spearman and Kärber [19,20].

### 2.4. Post-Treatment of the Carriers with Emesvirus Zinderi MS2

The carrier coated with the phage suspension received a different type of post-treatment. Similar to the processing mentioned above, the carriers were rinsed in 1 mL of LB medium and further titrated in LB medium. This approach was applied to prepared agar plates. For this purpose, the Petri dishes containing the LB–agar were covered with a suspension of *E. coli* and soft agar (1:10, 7 mL). After solidification of the soft agar, 10 µL from the titration approach (10^1^–10^6^) was dropped to the agar plate in triplicates. After 24 h (37 °C, 95% RH), plaques on the agar plate indicated the presence of infectious bacteriophages. The titer was calculated as described above.

### 2.5. Ultraviolet C (UV-C) Radiation

UV-C LED radiators with a wavelength of 233 nm or 275 nm (Ferdinand Braun Institute, Berlin, Germany, ams OSRAM Regensburg, Regensburg, Germany) were used to investigate the virucidal effect of UV-C radiation. The Full Width at Half Maximum (FWHM) of the 233 nm UV-C source was about 12 nm [6,21], and that of the 275 nm UV-C source was about 11.3 nm. The distance between the radiation source and the sample was set as 5 cm. The radiator with the 275 nm wavelength worked with an irradiance of 0.24 mW/cm^2^. The maximum dose set was 600 mJ/cm^2^. With regard to irradiation with the 233 nm radiator, an irradiance of 0.11–0.14 mW/cm^2^ was used. The irradiance was checked before every experiment using a calibrated sensor [6,16,17]. The LEDs were cooled during use to avoid overheating. This also maintained a stable intensity over the irradiation period. Irradiance was adjusted using an integrated dimmer.

The temperature of the carriers was measured over the test period and was not increased by any of the radiators.

In addition, a mercury radiator with a 254 nm wavelength that had an irradiation intensity of 0.50 mW/cm^2^ was used as the reference. For the inactivation kinetics, the intensity was reduced to 0.15 mW/cm^2^.

### 2.6. Statistical Analysis

The weighted mean was calculated by weighting the individual values by their errors:

$$\bar{x}=\frac{\sum_{i=1}^{n}p_{i}x_{i}}{\sum_{i=1}^{n}p_{i}}$$

The internal and external consistency were calculated according to the following formula:

$$\mathrm{Internal}\;\mathrm{consistency}:\;s_{int}^{2}=\frac{1}{\sum_{i=1}^{n}\frac{1}{s_{i}^{2}}}$$

$$\mathrm{External}\;\mathrm{consistency}:\;s_{ext}^{2}=\frac{1}{n-1}\frac{\sum_{i=1}^{n}{p_{i}(x_{i}-\bar{x})}^{2}}{\sum_{i=1}^{n}p_{i}}$$

where *n* is the number of test series, *x_i_* is the mean of each test series, *s_i_* is its standard deviation, and *p_i_* = 1/*s_i_*^2^. To avoid specifying an error that was too small, the larger value was specified as the weighted error [22].

For comparison of the virus inactivation using the different FCS contents of the cell culture medium, a paired t-test was used. For comparing the impact of the irradiance, 2-way ANOVA following Dunnett’s multiple comparison was used. To analyze the effects of varying the irradiance while the dose remained constant, Pearson’s correlation coefficient (r) was calculated.

## 3. Results

### 3.1. Inactivation Kinetics Using UV-C Radiation

**Figure 2:**
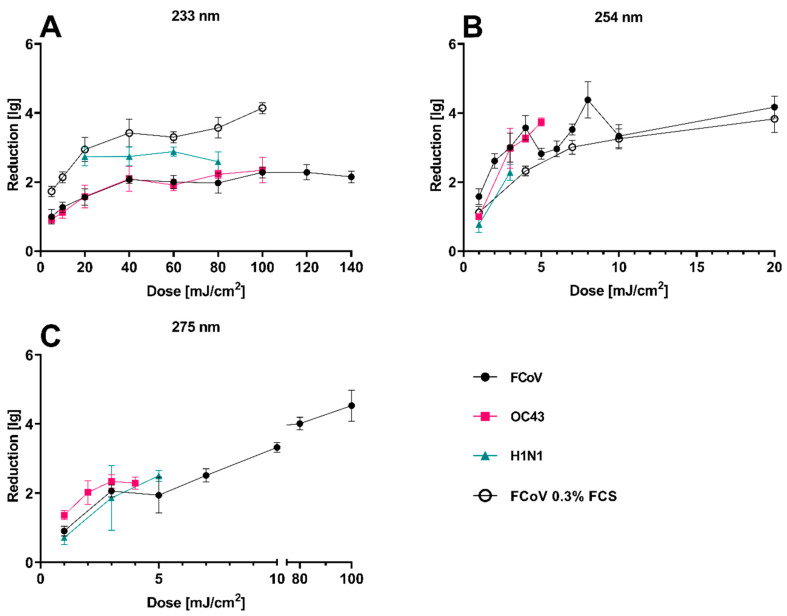
Dose–response curve of FCoV, OC43, and H1N1 treated with irradiation with different doses of 233 nm (**A**), 254 nm (**B**), and 275 nm (**C**), and the inactivation kinetics of FCoV with 2% and 0.3% FCS in cell culture medium for 233 nm and 254 nm radiation on steel carriers. Increasing the dose led to increased inactivation of the viruses in the carrier test. N = 3–7, weighted mean ± internal/external consistency.

The inactivation kinetics of FCoV, HCoV, and H1N1 using UV-C radiation on steel carriers were studied with all three wavelengths (233 nm, 254 nm, 275 nm, Figure 2). With higher doses, the inactivation increased. The required 4 lg inactivation [23] was not achieved for all three viruses using 233 nm radiation with a maximum dose of 140 mJ/cm^2^. Application of 254 nm UV-C radiation resulted in 4 lg inactivation for FCoV using a dose of 8 mJ/cm^2^. Using 275 nm radiation, a 4 lg inactivation was achieved for FCoV after an application of 80 mJ/cm^2^.

### 3.2. Effect of FCS Concentration on Virus Inactivation

Reducing the FCS content (0.3%) of the cell culture medium resulted in a significantly higher inactivation efficacy in comparison to the medium with 2% FCS for 233 nm radiation (mean of differences = −1.3 ± 0.39, *p* = 0.0001, Figure 2A). For 254 nm irradiation, no significant difference was found (mean of differences = 0.53 ± 0.44, *p* = 0.0538, Figure 2B).

### 3.3. Sensitivity of Viral Strains and Necessary Dose for 4 lg Inactivation

The sensitivity of the different viral strains to 233 nm irradiation varied (Table 2). A dose of 80 mJ/cm^2^ is considered skin-compatible [16]. Regarding the virus inactivation with 80 mJ/cm^2^ (233 nm), the necessary inactivation of 4 lg [23,24] was achieved solely for PV-1 on steel carriers. In comparison, an inactivation of only 3.48 or 3.32 lg was observed for SARS-CoV-2 or FCoV, respectively. There was no correlation of the inactivation efficacy with the genome type or the existence of an envelope. Comparing the carrier materials, there was also no general correlation.

**Figure 3:**
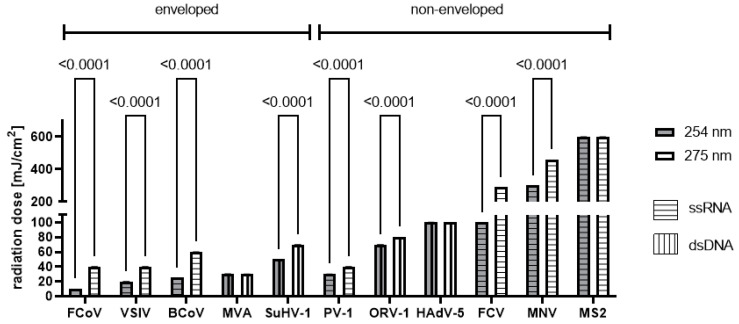
Comparison of the minimal radiation dose for a ≥4 lg inactivation at 254 nm and 275 nm on steel carriers.

Significantly higher minimal radiation doses were necessary to achieve an effective inactivation of 4 lg with the 275 nm radiator compared to the 254 nm mercury lamp (Figure 3, Supplementary Tables S2 and S3) for most of the tested viruses (*p* < 0.0001). Only HAdV-5, MVA, and MS2 required comparable radiation doses for effective inactivation with both wavelengths. With regard to the genome, no discernible difference could be observed between double-stranded and single-stranded or RNA or DNA viruses. Differences due to the carrier material were not observed (Supplementary Tables S3–S5).

### 3.4. Influence of Irradiance and Carrier Material by Using a 233 nm Radiator

**Figure 4:**
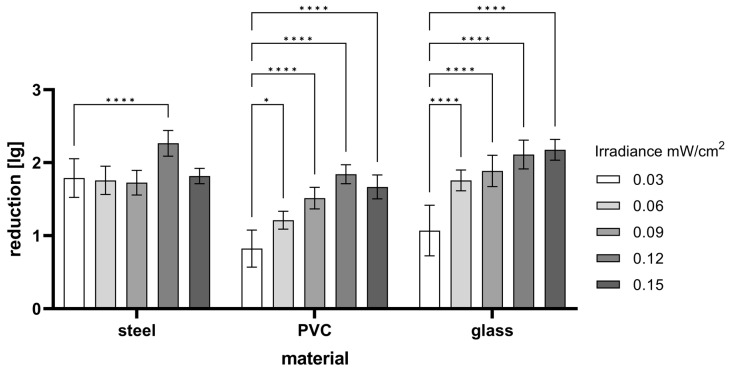
Effect of irradiance on virus inactivation (FCoV) on different materials when applying 20 mJ/cm^2^ (233 nm), with different irradiances resulting in different treatment times. Weighted mean ± internal/external consistency. * *p* < 0.05, **** *p* < 0.0001.

Regarding the irradiance level, the virus inactivation on steel carriers was not affected (r = 0.3982, *p* = 0.5068), except for 0.12 mW/cm^2^, which had detectable inactivation effects (Figure 4). Using glass (r = 0.9190, *p* = 0.0274) and PVC (r = 0.9113, *p* = 0.0313) carriers, increasing the irradiance increased the inactivation when applying identical doses of UV-C radiation. An irradiance of 0.15 mW/cm^2^ did not increase the effects in comparison to 0.12 mW/cm^2^.

## 4. Discussion

The most prominent challenge posed by respiratory virus infections is their aerogenic and inhalative transmission indoors. This is because—apart from mouth–nose protection, possibly supported by ventilation, filtration, and social distancing—there are no protective measures available to exclude the room air as a transmitter. Thus, new tools for the prevention of airborne viral infections are needed. The efficacy of 254 nm radiation in inactivating viruses, e.g., SARS-CoV-2, i.a., has been attested many times [25,26,27,28,29,30,31,32,33,34]. Although UV-C radiation of a 254 nm wavelength emitted by mercury vapor lamps is used for air decontamination, this radiation wavelength is considered potentially damaging for human skin, and because they contain mercury, the lamps themselves are ecologically questionable.

Two methods for the prevention of infection may be of interest: (1) the treatment of surfaces or room air by 275 nm LEDs as an environmentally friendly alternative to 254 nm lamps; and (2) the treatment of the mouth and throat of humans to reduce transmission via aerosols by inactivating viruses directly at their place of origin with a peak wavelength of, e.g., 233 nm in special cases, such as during dental treatment, as a protective measure for operating staff. The tolerance of mucous membranes to this wavelength has already been demonstrated [35] and can also be concluded from the lower penetration depth vs. longer wavelengths due to absorption. However, the virucidal efficacy has yet to be demonstrated. In vivo, of course, saliva will absorb the radiation to some extent and reduce the effectiveness of the radiation. The absorption of radiation by saliva is comparable to the absorption of the 3% FCS medium used in the present study (Supplementary Figure S1). Furthermore, the absorption also protects the underlying cells, so the use of higher doses seems possible; this must, however, still be proven in detail in vitro. A higher wavelength, e.g., 275 nm, is more efficient in terms of virus inactivation. Thus, it may be an alternative for room disinfection.

Since 275 nm radiation is not skin-compatible, it is not recommended for use when persons are present and who may be directly exposed to the radiation. Radiation at 233 nm is more skin-compatible than radiation at 254 nm or 275 nm, and it can hence be used in direct contact with persons and their skin. However, for a use as room disinfectant, its efficacy is too low.

From a methodological point of view, in the present study, the virus inactivation was analyzed on surfaces as a worst-case scenario because it can be assumed that viruses are more difficult to inactivate on surfaces after drying than in room air or in suspension. To determine the effect on virus inactivation, it is also feasible to simulate the influence of different loads/matrices on different surfaces. The results allow conclusions to be drawn regarding the suitability for surface disinfection.

Not only was the effectiveness against viruses transmitted via aerosols or particles in the air (airborne) tested, but the effectiveness against viruses transmitted via inanimate objects (fomite-borne) was also tested, because the transmission via the latter is prevented by surface disinfection, for which purpose UV-C can also be used. To compare the efficacy against enveloped and non-enveloped viruses, both types of viruses were included in this study. However, only ssRNA and dsDNA viruses were used.

### 4.1. Far-UV-C Radiation at 233 nm

Data from the literature demonstrate the virucidal activity of far-UV-C radiation. Buonanno et al. reported the virucidal activity of 222 nm radiation [36]. However, the required 4 lg inactivation for use as an antiseptic agent [23,24,37] was not reached, and the necessary doses were high in these experiments as well as in other studies [38,39,40,41]. For 233 nm radiation, no published data are available regarding its virucidal activity. However, data regarding the bactericidal activity of 233 nm radiation [16,17] and the virucidal activity of 222 nm radiation [36] are promising and provide evidence of the potential virucidal efficacy of 233 nm wavelength radiation.

The skin and mucosa compatibility of 233 nm radiation emitted by LED has already been confirmed for certain doses [16,35]. A maximum of 60–80 mJ/cm^2^ was revealed to be skin-tolerable. In the present study, the virucidal activity of this far-UV-C wavelength was examined. For studying the inactivation kinetics of low doses of far-UV-C radiation, three different viruses (FCoV, HCoV, and H1N1) were used. With increasing doses, increasing inactivation was observed. However, the increase declined for higher doses, and doses > 40–60 mJ/cm^2^ did not lead to further significant increases in inactivation (lg reduction). This non-linear behavior might have been a result of shielding effects that become more pronounced with higher doses, since by then, all non-protected viruses had already been inactivated. Furthermore, high amounts of viruses in small volumes might lead to agglomeration of the viruses and, thus, to a self-shielding effect.

When reducing the protein load by decreasing the FCS content to 0.3%, the inactivation efficacy increased due to a lower absorption of the radiation by non-target molecules, as shown previously [16]. This effect mainly occurred at 233 nm, and it occurred less often at 254 nm radiation, because the longer wavelength chiefly interacted with DNA/RNA and, to a lesser degree, with the proteins (Supplementary Figure S1).

Next, 12 viruses were used to detect the effect of 80 mJ/cm^2^ as a skin-tolerable dose. A 4 lg inactivation was only achieved for PV-1. It must be mentioned that in order to demonstrate a 4 lg reduction, the initial titer has to be high enough. For H1N1, this initial titer was too low, and it furthermore possessed a relatively high limit of detection, such that a 4 lg inactivation was not achievable. Moreover, a carrier test with a dried virus suspension was used. As shown by Buonanno et al. and Welch at al., higher inactivation rates of up to 3 lg with low doses of 1.2–1.7 mJ/cm^2^ are possible when using aerosolized viruses [36,42]. However, the requirement of 4 lg is set by, e.g., DIN EN regulations [23,24] without epidemiological evidence. For example, the inhalative infectious dose for influenza A (H3N2) and for different serotypes of influenza B is >10^3^ TCID_50_; for RSV (conjunctival), it is 10^5^ TCID_50_; for adenovirus (oral), it is >10^6^ TCID_50_; and for different serotypes of influenza A (H1N1), it is even >10^6^–>10^7^ TCID_50_ [43]. This means that for these airborne viruses, a reduction of 4 lg is not necessarily required to prevent inhalative transmission.

Statistically significant differences between the inactivation efficacy on the different materials and between the viruses were detected. However, these differences do not follow a pattern; therefore, no conclusion can be drawn. Also, no patterns regarding single- and double-stranded DNA/RNA were present. In contrast, Kowalski et al. and Gerba et al. revealed double-stranded viruses to be more resistant towards UV radiation than single-stranded viruses [44,45,46] due to their higher purine content, since purines are more resistant to radiation than pyrimidines [47]. The differences in the present data could be attributed to the shift from mainly DNA/RNA being the target molecules to also include protein damage by using a 233 nm peak wavelength. Also, the use of viruses either in suspension or dried on a surface could have affected the outcome.

The treatment time is especially of interest for use on skin or mucosa. This was the reason for testing the effect of increasing the irradiance with a constant dose of 20 mJ/cm^2^ (Figure 4). An increasing virucidal effect was observed with the carriers made of glass and PVC when the irradiance was increased, and thus the treatment time was shortened until a maximum was reached at 0.12 mW/cm^2^. These effects could have arisen because the high intensity caused a great deal of damage at once, which led to the inactivation of the viruses, whereas a low dose caused little damage. Stable bonds of DNA/RNA or proteins would thus have not been damaged, even with a longer treatment at a low dose, and the overall inactivation would hence have remained lower. On the steel carriers, these effects could have possibly been weakened by reflection from the carrier surface.

In contrast, Kitagawa et al. did not observe an effect of the irradiance (0.003–0.1 mW/cm^2^) when applying 222 nm radiation (doses 1–3 mJ/cm^2^) on a polystyrene surface [38,39]. The differences in the current data might have occurred due to the differences in the doses used and the different effects of the 222 nm and 233 nm radiation on the DNA/RNA and proteins that may have altered the outcome of the irradiation. The 222 nm radiation was absorbed more strongly by the proteins than the 233 nm radiation, reducing its efficacy. This might have also resulted in differences regarding the effects of the irradiance. Furthermore, in the present study, differences between the materials occurred. Kitagawa et al. used polystyrene plates that might have a different effect than that of the material used in the current study. In this study, increasing inactivation with increasing irradiance was also not observed when the steel carriers were used, which could have been due to the reflection of the radiation.

### 4.2. Near-UV-C Radiation at 275 nm

As is the case for 254 nm radiation, radiation with peak wavelength of 275 nm cannot be used in the presence of humans. However, 275 nm radiation can be emitted by LEDs, which are more environmentally friendly and show greater durability as well as less energy consumption [48,49,50]. Furthermore, there is no risk of contamination from toxic mercury when working with LED radiators [51]. The virucidal activity of 275 nm radiation against HCoV 229E was demonstrated by Singh et al., revealing a 4.94 lg inactivation after the application of 20.61 mJ/cm^2^ radiation [52]. This inactivation was higher than that detected in the present study for the different coronaviruses (Supplementary Table S3), which can be explained by the smaller volume used and differences in the protein concentration in the medium. Differences in susceptibility also occurred between the coronaviruses used, making a comparison to HCOV 229E more difficult.

In the present study, the virucidal activity of the 275 nm radiation increased with increasing doses. For most virus strains, the required doses for 4 lg inactivation were higher with the 275 nm radiation than with the 254 nm radiation for enveloped as well as non-enveloped viruses. However, it must be borne in mind that the irradiance of the lamps was not equal; that of the 254 nm lamp was higher than that of the 275 nm radiator. Similar to the effects we observed at 233 nm with varying intensity, this may mean that the effectiveness is not comparable (or only to a very limited extent) due to the different intensities.

In comparison to the 254 nm radiation, the UV-C radiation with a 275 nm wavelength was less efficient in terms of virus inactivation. In the case of enveloped viruses, the dose needed to be 20–30 mJ/cm^2^ higher (FCoV, VSIV, BCoV, SuHV-1) when using the 275 nm radiation. For non-enveloped viruses, these differences were more pronounced, since the dose needed to be 160–180 mJ/cm^2^ higher (FCV, MNV). However, 275 nm radiation produced by LEDs has advantages regarding environmental friendliness and durability, thus making it a good alternative for use in air, water, and surface disinfection systems.

For non-enveloped ssRNA viruses, the necessary doses for 4 lg inactivation were the highest, with some exceptions. In general, non-enveloped viruses are often more chemo-resistant [53] and more environmentally stable than enveloped viruses [54], which agrees with our data regarding inactivation via 275 nm radiation. For enveloped viruses, the necessary dose for 4 lg inactivation was very similar. These results are in accordance with data published by Duc Canh et al., who stated that the inactivation was independent of the capsid or the lipid bilayer but might depend on the genomic properties, such as DNA/RNA length [55]. However, the wavelengths used (265 nm, 280 nm) might have properties different from those of 275 nm radiation, since absorption via DNA/RNA and proteins depends on the wavelength, and thus so does damage/inactivation.

## 5. Conclusions

Far-UV-C radiation has virucidal activity and can be an additional measure for reducing virus transmission. In order to interrupt airborne and fomite-borne infections, when inactivating viruses, the surrounding medium is pivotal to the efficacy of the radiation. Similar in vitro results have already been obtained by Zwicker et al. and Sicher et al. [16,17]. Furthermore, as previously shown in vitro for bacteria [16,17], the strain or genus of viruses is not crucial in terms of the efficacy of radiation. However, non-enveloped viruses are often harder to inactivate than enveloped viruses.

Unfortunately, skin-tolerable doses of far-UV-C radiation are not sufficient for reducing viruses > 4 lg. However, a 4 lg inactivation might not be necessary for the prevention of virus transmission. Furthermore, the use of skin-tolerable doses might still be of benefit in reducing infection transmission as an additional measure. To this extent, UV-C might be especially applicable as a prophylactic measure. Near-UV-C radiation (275 nm) is very similar to 254 nm radiation in terms of its virucidal activity, but it has the advantage of being more environmentally friendly.

UV-C irradiation is an effective method of virus inactivation. Peak wavelengths of 233 nm and 275 nm have now become available through recent advancements in UV-C-LED technology and could possibly lead to new applications. UV-C radiation with a 233 nm peak wavelength was able to inactivate nearly 4 lg of FCoV (0.3% FCS) when using a skin-compatible dose of 80 mJ/cm^2^. This means that it could be a measure for the prophylaxis of airborne infections by treatment of the mouth or throat (for instance) to reduce viral transmission by aerosols as an additional measure to, e.g., wearing face masks or gargling. Since skin compatibility is not necessary when irradiating rooms in the absence of persons, a 4 lg reduction, as required by DIN EN17272, i.a., using 275 nm LED radiators is possible. Thus, these LED radiators are an environmentally friendlier alternative to 254 nm radiation for use in room disinfection.

## Figures and Tables

**Table 1:** Sources of supply of the viruses used and appropriate cells for the inactivation experiments, their properties regarding DNA/RNA, and infection titers.

Virus	Properties	Cell Culture/Host Name	Initial Titer (lg)
HAdV-5 (ATCC, VR-1516)	dsDNA, non-enveloped	A 549 (CCLV-RIE 1035) lung (*Homo sapiens*)	7.58 ± 0.15
BCoV (strain “Nebraska”, RVB-0003)	ssRNA, enveloped	PT (CCLV-RIE 0011) sheep (*Ovis orientalis aries*)	7.47 ± 0.21
FCoV (strain “Munich”, RVB-1259)	ssRNA, enveloped	CRFK (CCLV-RIE 0115) cat kidney (*Felis catus*)	5.99 ± 0.98/ 7.47 ± 0.27 *
MNV (strain “S99”, RVB-0651)	ssRNA, non-enveloped	RAW 264.7(CCLV-RIE 0996) Ascites of mouse strain BALB/c (*Mus musculus*)	9.25 ± 0.18
FCV (strain “F-9”, RVB-0208)	ssRNA, non-enveloped	KE-R (CCLV-RIE 0138) cat fetus (*Felis catus*)	8.63 ± 0.27
PV-1 (Sabin, vaccine strain “LSc-2ab”, RVB-1260)	ssRNA, non-enveloped	BHM (CCLV-RIE 0136) African green monkey kidney (*Chlorocebus sabaeus*)	6.47 ± 0.17
MS2 (DSM number: 13767)	ssRNA, non-enveloped	*E. coli* (DSM number: 5695) Lederberg W1485	8.47 ± 0.17
MVA (RVB-1332)	dsDNA, enveloped	BHK-21 (CCLV-RIE 0179) hamster kidney (*Mesocricetus auratus*)	7.02 ± 0.15
ORV-1 (strain “Erik”, RVB-0391)	dsDNA, non-enveloped	ST (CCLV-RIE 0606) domestic pig testis (*Sus scrifa domestica*)	7.69 ± 0.18
SARS-CoV-2 (strain “BavPat1/2020”)	ssRNA, enveloped	VERO E6 (CCLV-RIE 0929) African green monkey kidney (*Chlorocebus sabaeus*)	6.63 ± 0.17
SuHV-1 (strain “Kaplan”, RVB-0574)	dsDNA, enveloped	ST (CCLV-RIE 0606) domestic pig testis (*Sus scrifa domestica*)	7.30 ± 0.22
VSIV (RVB-0030)	ssRNA, enveloped	KOP-R (CCLV-RIE 0244) domestic cattle esophagus (*Bos primigenius taurus*)	7.51 ± 0.16
HCoV OC43 (ATCC, VR-1558)	ssRNA, enveloped	MRC-5 (ATCC, CCL-171) human lung fibroblast	6.01 ± 1.19
H1N1 (ATCC, VR-1469, strain “A/PR/8/34”)	ssRNA, enveloped	MDCK (CLS, NBL-2) canine, kidney, epithelium	5.37 ± 0.55

ATCC—American Type Culture Collection, RVB—Virus Collection of the Friedrich Loeffler Institute (Riemser Virusbank), CCLV-RIE—Cell Culture Collection of the Friedrich Loeffler Institute (Riemser Zellbank), DSM—Leibniz Institute (German Collection of Microorganisms and cell culture GmbH), CLS—cell line service; * lower titer stock was used for the following tests: inactivation kinetics, effects of FCS concentration, influence of irradiance, and carrier material. All other experiments (inactivation by 233 nm with 80 mJ/cm^2^, necessary dose for 4 lg inactivation) were performed with the higher titer stock.

**Table 2:** Values of lg inactivation after irradiation of various viruses with 80 mJ/cm^2^ (233 nm), n ≥ 3, weighted mean ± weighted SD.

		Virus	Inactivation [lg ± SD] Stainless Steel	Inactivation [lg ± SD] PVC	Inactivation [lg ± SD] Glass	Statistical Significance Steel vs. PVC	Statistical Significance Steel vs. Glass	Statistical Significance PVC vs. Glass
Enveloped	ssRNA	SARS-CoV-2	3.48 ± 0.43	2.88 ± 0.18	2.96 ± 0.26	0.0030	0.0116	n.s.
		FCoV	3.32 ± 0.19	2.77 ± 0.30	2.79 ± 0.33	0.0071	0.0099	n.s.
		VSIV	2.83 ± 0.13	2.00 ± 0.16	2.61 ± 0.32	<0.0001	n.s.	0.0025
		BCoV	1.56 ± 0.09	1.40 ± 0.10	1.67 ± 0.26	n.s.	n.s.	0.2810
	dsDNA	MVA	1.61 ± 0.07	2.76 ± 0.09	2.18 ± 0.28	<0.0001	0.0051	0.0043
		SuHV-1	1.32 ± 0.11	1.73 ± 0.11	2.22 ± 0.32	n.s.	<0.0001	0.0185
Non-enveloped	dsDNA	PV-1	4.28 ± 0.18	2.48 ± 0.17	2.85 ± 0.11	<0.0001	<0.0001	n.s.
		ORV-1	1.49 ± 0.08	1.79 ± 0.08	1.84 ± 0.13	n.s.	n.s.	n.s.
		HAdV-5	2.88 ± 0.37	2.76 ± 0.17	2.92 ± 0.14	n.s.	n.s.	n.s.
	ssRNA	MS 2	1.50 ± 0.12	0.44 ± 0.25	1.34 ± 0.29	<0.0001	n.s.	<0.0001
		FCV	3.28 ± 0.30	2.51 ± 0.15	3.18 ± 0.19	0.0001	n.s.	0.0008
		MNV	1.73 ± 0.28	1.46 ± 0.11	1.59 ± 0.08	n.s.	n.s.	n.s.

n.s.: not significant.

## Data Availability

The data that support the findings of this study are available from the corresponding author.

## Supplementary Materials

The following supporting information can be downloaded at: https://www.mdpi.com/article/10.3390/v16121904/s1, Figure S1: Absorption of radiation by cell culture medium without and with FCS as well as natural and artificial saliva that was used in former studies; Table S1: Used media conditions for the multiplication of the viruses and the bacteriophage; Table S2: Results of UV C irradiation, 254 nm (mercury lamp), stainless steel; Table S3: Results of UV C irradiation, 275 nm (LED radiator), stainless steel; Table S4: Results of UV C irradiation, 275 nm (LED radiator), PVC; Table S5: Results of UV C irradiation, 275 nm (LED radiator), glass; Table S6: Results of UV C irradiation, 233 nm (LED radiator), stainless steel; Table S7: Results of UV C irradiation, 233 nm (LED radiator), PVC; Table S8: Results of UV C irradiation, 233 nm (LED radiator), glass.
