# Supplementary material for: Studies on the Virucidal Effects of UV-C of 233 nm and 275 nm Wavelengths
Source: Viruses. 2024 Dec 11;16(12):1904. doi: 10.3390/v16121904 (PMC11680280; doi:10.3390/v16121904)

viruses

Table S1: Used media conditions for the multiplication of the viruses and the bacteriophage.

Virus	medium
HAdV-5	Ham's F-12 Nutrient Mix powder with L-glutamine, IMDM powder with L-glutamine, $NaHCO_3$
BCoV (RVB-0003)	MEM (E), MEM (HBSS), $NaHCO_3$, sodium pyruvate, NEA
FCoV (RVB-1259)	MEM (E), MEM (HBSS), $NaHCO_3$, sodium pyruvate, NEA
MNV (RVB-0651)	MEM (E), MEM (HBSS), $NaHCO_3$, sodium pyruvate, NEA
FCV (RVB-0208)	MEM (H), MEM (HBSS), $NaHCO_3$
Human Poliovirus type 1 (Sabin, vaccine strain "LSc-2ab", RVB-1260)	Ham's F-12 Nutrient Mix powder with L-glutamine, IMDM powder with L-glutamine, $NaHCO_3$
MS2 (DSM number: 13767)	LB-medium (Roth, Karlsruhe, Germany)
MVA (RVB-1332)	MEM (E), MEM (HBSS), $NaHCO_3$, sodium pyruvate, NEA
ORV-1 (RVB-0391)	Ham's F-12 Nutrient Mix powder with L-glutamine, IMDM powder with L-glutamine, $NaHCO_3$
SARS-CoV-2 (strain "BavPat1/2020")	MEM (E), MEM (HBSS), $NaHCO_3$, sodium pyruvate, NEA
SuHV-1 (RVB-0574)	Ham's F-12 Nutrient Mix powder with L-glutamine, IMDM powder with L-glutamine, $NaHCO_3$
VSIV (RVB-0030)	MEM (H), MEM (HBSS), $NaHCO_3$
HCoV OC43	MEM (E), MEM (HBSS), $NaHCO_3$, sodium pyruvate, NEA
Influenza A virus (H1N1, strain "A/PR/8/34")	DMEM/F12, 0.5% FCS, 1.5% Panexin CD

Table S2: Results of UV C irradiation, 254 nm (mercury lamp), stainless steel, n=3, green= inactivation including internal and external consistency ≥ 4 lg, n.d.=not determined

radiationdose [mJ/cm²]	BCoV	FCoV	VSIV	SuHV-1	MVA	MS 2	PV-1	FCV	MNV	ORV-1	HAdV-5
	lg inactivation (weighting mean ± internal consistency ± external consistency)										
5	n.d.	3,48<br>±0,13<br>±0,20	n.d.	n.d.	n.d.	n.d.	n.d.	n.d.	n.d.	n.d.	n.d.
10	n.d.	4,25<br>±0,08<br>±0,10	2,98<br>±0,09<br>±0,08	n.d.	n.d.	n.d.	n.d.	n.d.	n.d.	n.d.	n.d.
20	3,92<br>±0,09<br>±0,20	4,63<br>±0,12<br>±0,10	4,21<br>±0,08<br>±0,10	n.d.	3,71<br>±0,10<br>±0,09	n.d.	n.d.	n.d.	n.d.	n.d.	n.d.
25	4,29<br>±0,14<br>±0,04	n.d.	4,72<br>±0,13<br>±0,18	n.d.	n.d.	n.d.	3,91<br>±0,11<br>±0,05	n.d.	n.d.	n.d.	n.d.
30	4,71<br>±0,07<br>±0,22	n.d.	n.d.	n.d.	4,39<br>±0,11<br>±0,39	n.d.	4,20<br>±0,09<br>±0,05	n.d.	n.d.	n.d.	n.d.
35	n.d.	n.d.	n.d.	n.d.	n.d.	n.d.	4,39<br>±0,08<br>±0,11	n.d.	n.d.	n.d.	n.d.
40	n.d.	n.d.	n.d.	3,61<br>±0,09<br>±0,23	4,60<br>±0,12<br>±0,22	n.d.	n.d.	n.d.	n.d.	n.d.	n.d.
50	n.d.	n.d.	n.d.	4,44<br>±0,10<br>±0,10	n.d.	n.d.	n.d.	n.d.	n.d.	n.d.	n.d.
60	n.d.	n.d.	n.d.	4,70<br>±0,09<br>±0,12	n.d.	n.d.	n.d.	n.d.	n.d.	3,94<br>±0,10<br>±0,20	n.d.
70	n.d.	n.d.	n.d.	n.d.	n.d.	n.d.	n.d.	n.d.	n.d.	4,19<br>±0,08<br>±0,09	n.d.
80	n.d.	n.d.	n.d.	n.d.	n.d.	n.d.	n.d.	n.d.	n.d.	4,45<br>±0,08<br>±0,14	n.d.

90	n.d.	n.d.	n.d.	n.d.	n.d.	n.d.	n.d.	3,44<br>±0,10<br>±0,16	n.d.	n.d.	3,04<br>±0,10<br>±0,52
100	n.d.	n.d.	n.d.	n.d.	n.d.	n.d.	n.d.	4,33<br>±0,09<br>±0,15	n.d.	n.d.	4,26<br>±0,08<br>±0,21
110	n.d.	n.d.	n.d.	n.d.	n.d.	n.d.	n.d.	4,59<br>±0,10<br>±0,19	n.d.	n.d.	4,86<br>±0,09<br>±0,03
120	n.d.	n.d.	n.d.	n.d.	n.d.	n.d.	n.d.	n.d.	n.d.	n.d.	n.d.
290	n.d.	n.d.	n.d.	n.d.	n.d.	n.d.	n.d.	n.d.	3,77<br>±0,07<br>±0,08	n.d.	n.d.
300	n.d.	n.d.	n.d.	n.d.	n.d.	n.d.	n.d.	n.d.	4,31<br>±0,10<br>±0,15	n.d.	n.d.
310	n.d.	n.d.	n.d.	n.d.	n.d.	n.d.	n.d.	n.d.	4,45<br>±0,12<br>±0,12	n.d.	n.d.
550	n.d.	n.d.	n.d.	n.d.	n.d.	3,97<br>±0,08<br>±0,03	n.d.	n.d.	n.d.	n.d.	n.d.
600	n.d.	n.d.	n.d.	n.d.	n.d.	4,18<br>±0,09<br>±0,18	n.d.	n.d.	n.d.	n.d.	n.d.

Table S3: Results of UV C irradiation, 275 nm (LED radiator), stainless steel, n=3, green= inactivation including internal and external consistency ≥ 4 lg, n.d.=not determined

radiatondose [mJ/cm²]	BCoV	FCoV	SARS CoV-2	VSIV	SuHV-1	MVA	MS 2	PV-1	FCV	MNV	ORV-1	HAdV-5
	lg inactivation (weighting mean ± internal consistency ± external consistency)											
5	n.d.	n.d.	n.d.	n.d.	n.d.	n.d.	n.d.	n.d.	n.d.	n.d.	n.d.	n.d.
10	n.d.	n.d.	3,76<br>±0,08<br>±0,11	n.d.	n.d.	3,51<br>±0,11<br>±0,17	n.d.	n.d.	n.d.	n.d.	n.d.	n.d.
20	n.d.	3,33<br>±0,12<br>±0,25	4,39<br>±0,10<br>±0,14	3,47<br>±0,11<br>±0,07	n.d.	4,04<br>±0,08<br>±0,20	n.d.	n.d.	n.d.	n.d.	n.d.	n.d.
30	n.d.	4,09<br>±0,11<br>±0,48	4,66*<br>±0,07<br>±0,02	4,04<br>±0,10<br>±0,17	n.d.	4,62<br>±0,10<br>±0,14	n.d.	3,60<br>±0,08<br>±0,13	n.d.	n.d.	n.d.	n.d.
40	2,90<br>±0,13<br>±0,24	4,31<br>±0,10<br>±0,09	n.d.	4,38<br>±0,08<br>0,20	n.d.	4,89<br>±0,06<br>±0,17	n.d.	4,30<br>±0,07<br>±0,12	n.d.	n.d.	n.d.	n.d.
50	3,67<br>±0,12<br>±0,20	4,62<br>±0,10<br>±0,23	n.d.	4,58<br>±0,11<br>0,12	n.d.	n.d.	n.d.	4,45*<br>±0,09<br>±0,08	n.d.	n.d.	n.d.	n.d.
60	4,50<br>±0,11<br>±0,09	n.d.	n.d.	n.d.	3,98<br>±0,11<br>±0,04	n.d.	n.d.	n.d.	n.d.	n.d.	n.d.	n.d.
70	4,77<br>±0,10<br>±0,19	n.d.	n.d.	n.d.	4,51<br>±0,07<br>±0,20	n.d.	n.d.	n.d.	n.d.	n.d.	3,21<br>±0,10<br>±0,43	n.d.
80	n.d.	n.d.	n.d.	n.d.	4,39<br>±0,12<br>±0,11	n.d.	n.d.	n.d.	n.d.	n.d.	4,09<br>±0,07<br>±0,03	n.d.
90	n.d.	n.d.	n.d.	n.d.	4,36<br>±0,09<br>±0,24	n.d.	n.d.	n.d.	n.d.	n.d.	4,56<br>±0,07<br>±0,09	3,51<br>±0,08<br>±0,16
100	n.d.	n.d.	n.d.	n.d.	4,97<br>±0,08<br>±0,11	n.d.	n.d.	n.d.	n.d.	n.d.	n.d.	4,09<br>±0,08<br>±0,07
110	n.d.	n.d.	n.d.	n.d.	n.d.	n.d.	n.d.	n.d.	n.d.	n.d.	n.d.	4,72<br>±0,09<br>±0,17

180	n.d.	n.d.	n.d.	n.d.	n.d.	n.d.	n.d.	n.d.	n.d.	n.d.	4,46<br>±0,08<br>±0,15	n.d.
230	n.d.	n.d.	n.d.	n.d.	n.d.	n.d.	n.d.	n.d.	3,30<br>±0,08<br>±0,31	n.d.	n.d.	n.d.
240	n.d.	n.d.	n.d.	n.d.	n.d.	n.d.	n.d.	n.d.	3,40<br>±0,07<br>±0,25	n.d.	n.d.	n.d.
250	n.d.	n.d.	n.d.	n.d.	n.d.	n.d.	n.d.	n.d.	3,23<br>±0,10<br>±0,23	n.d.	n.d.	n.d.
280	n.d.	n.d.	n.d.	n.d.	n.d.	n.d.	n.d.	n.d.	3,43<br>±0,08<br>±0,06	n.d.	n.d.	n.d.
290	n.d.	n.d.	n.d.	n.d.	n.d.	n.d.	n.d.	n.d.	4,36<br>±0,09<br>±0,08	n.d.	n.d.	n.d.
300	n.d.	n.d.	n.d.	n.d.	n.d.	n.d.	n.d.	n.d.	4,54<br>±0,12<br>±0,18	n.d.	n.d.	n.d.
440	n.d.	n.d.	n.d.	n.d.	n.d.	n.d.	n.d.	n.d.	n.d.	3,61<br>±0,12<br>±0,06	n.d.	n.d.
450	n.d.	n.d.	n.d.	n.d.	n.d.	n.d.	n.d.	n.d.	n.d.	4,03<br>±0,12<br>±0,19	n.d.	n.d.
460	n.d.	n.d.	n.d.	n.d.	n.d.	n.d.	n.d.	n.d.	n.d.	4,14<br>±0,12<br>±0,02	n.d.	n.d.
470	n.d.	n.d.	n.d.	n.d.	n.d.	n.d.	n.d.	n.d.	n.d.	4,70<br>±0,16<br>±0,20	n.d.	n.d.
550	n.d.	n.d.	n.d.	n.d.	n.d.	n.d.	3,14<br>±0,09<br>±0,13	n.d.	n.d.	n.d.	n.d.	n.d.

600	n.d.	n.d.	n.d.	n.d.	n.d.	n.d.	4,12 ±0,11 ±0,07	n.d.	n.d.	n.d.	n.d.	n.d.

Table S4: Results of UV C irradiation, 275 nm (LED radiator), PVC, n=3, green= inactivation including internal and external consistency ≥ 4 lg, n.d.=not determined

radiation [mJ/cm²]	BCoV	FCoV	SARS CoV-2	VSIV	SuHV-1	MVA	MS 2	PV-1	FCV	MNV	ORV-1	HAdV-5
	lg inactivation (weighting mean ± internal consistency ± external consistency)											
5	n.d.	n.d.	n.d.	n.d.	n.d.	n.d.	n.d.	n.d.	n.d.	n.d.	n.d.	n.d.
10	n.d.	n.d.	n.d.	n.d.	n.d.	n.d.	n.d.	n.d.	n.d.	n.d.	n.d.	n.d.
20	n.d.	n.d.	n.d.	n.d.	n.d.	3,99 ±0,07 ±0,32	n.d.	n.d.	n.d.	n.d.	n.d.	n.d.
30	3,31 ±0,10 ±0,10	3,54 ±0,12 ±0,20	3,95 ±0,09 ±0,17	3,78 ±0,10 ±0,11	n.d.	4,21 ±0,07 ±0,46	n.d.	n.d.	n.d.	n.d.	n.d.	n.d.
40	4,32 ±0,12 ±0,29	4,88 ±0,09 ±0,23	4,15 ±0,08 ±0,48	4,54 ±0,06 ±0,54	n.d.	4,89 ±0,10 ±0,11	n.d.	n.d.	n.d.	n.d.	n.d.	n.d.
50	4,70 ±0,11 ±0,08	4,82 ±0,09 ±0,24	4,55* ±0,11 ±0,07	4,63 ±0,09±0,17	n.d.	n.d.	n.d.	n.d.	n.d.	n.d.	n.d.	n.d.
60	n.d.	n.d.	n.d.	n.d.	n.d.	n.d.	n.d.	3,92 ±0,10 ±0,05	n.d.	n.d.	n.d.	n.d.
70	n.d.	n.d.	n.d.	n.d.	3,33 ±0,11 ±0,20	n.d.	n.d.	4,19 ±0,08 ±0,12	n.d.	n.d.	n.d.	n.d.
80	n.d.	n.d.	n.d.	n.d.	3,66 ±0,11 ±0,12	n.d.	n.d.	4,55 ±0,08 ±0,17	n.d.	n.d.	3,43 ±0,07 ±0,17	n.d.
90	n.d.	n.d.	n.d.	n.d.	3,62 ±0,09 ±0,32	n.d.	n.d.	n.d.	n.d.	n.d.	n.d.	3,77 ±0,08 ±0,36
100	n.d.	n.d.	n.d.	n.d.	3,65 ±0,10 ±0,24	n.d.	n.d.	n.d.	n.d.	n.d.	n.d.	4,24 ±0,08 ±0,23
110	n.d.	n.d.	n.d.	n.d.	3,86 ±0,13 ±0,07	n.d.	n.d.	n.d.	n.d.	n.d.	n.d.	4,37 ±0,09 ±0,26
120	n.d.	n.d.	n.d.	n.d.	4,45 ±0,11 ±0,21	n.d.	n.d.	n.d.	n.d.	n.d.	n.d.	n.d.

130	n.d.	n.d.	n.d.	n.d.	4,49*<br>±0,08<br>±0,02	n.d.	n.d.	n.d.	n.d.	n.d.	n.d.	n.d.
170	n.d.	n.d.	n.d.	n.d.	n.d.	n.d.	n.d.	n.d.	n.d.	n.d.	3,56<br>±0,08<br>±0,16	n.d.
180	n.d.	n.d.	n.d.	n.d.	n.d.	n.d.	n.d.	n.d.	n.d.	n.d.	4,35<br>±0,08<br>±0,19	n.d.
190	n.d.	n.d.	n.d.	n.d.	n.d.	n.d.	n.d.	n.d.	n.d.	n.d.	4,67<br>±0,07<br>±0,26	n.d.
230	n.d.	n.d.	n.d.	n.d.	n.d.	n.d.	n.d.	n.d.	3,88<br>±0,11<br>±0,09	n.d.	n.d.	n.d.
240	n.d.	n.d.	n.d.	n.d.	n.d.	n.d.	n.d.	n.d.	4,39<br>±0,12<br>±0,22	n.d.	n.d.	n.d.
250	n.d.	n.d.	n.d.	n.d.	n.d.	n.d.	n.d.	n.d.	4,59<br>±0,15<br>±0,04	n.d.	n.d.	n.d.
290	n.d.	n.d.	n.d.	n.d.	n.d.	n.d.	n.d.	n.d.	4,73<br>±0,10<br>±0,16	n.d.	n.d.	n.d.
450	n.d.	n.d.	n.d.	n.d.	n.d.	n.d.	n.d.	n.d.	n.d.	3,28<br>±0,10<br>±0,10	n.d.	n.d.
460	n.d.	n.d.	n.d.	n.d.	n.d.	n.d.	n.d.	n.d.	n.d.	3,50<br>±0,12<br>±0,24	n.d.	n.d.
500	n.d.	n.d.	n.d.	n.d.	n.d.	n.d.	n.d.	n.d.	n.d.	3,67<br>±0,13<br>±0,17	n.d.	n.d.
550	n.d.	n.d.	n.d.	n.d.	n.d.	n.d.	3,57<br>±0,11<br>±0,06	n.d.	n.d.	4,36<br>±0,14<br>±0,29	n.d.	n.d.

600	n.d.	n.d.	n.d.	n.d.	n.d.	n.d.	4,00 ±0,07 ±0,07	n.d.	n.d.	4,69 ±0,11 ±0,07	n.d.	n.d.

Table S5: Results of UV C irradiation, 275 nm (LED radiator), glass, n=3, green= inactivation including internal and external consistency ≥ 4 lg, n.d.=not determined

radiation dose [mJ/cm^2]	BCoV	FCoV	SARS CoV-2	VSIV	SuHV-1	MVA	MS 2	PV-1	FCV	MNV	ORV-1	HAdV-5
	lg inactivation (weighting mean ± internal consistency ± external consistency)											
5	n.d.	n.d.	n.d.	n.d.	n.d.	n.d.	n.d.	n.d.	n.d.	n.d.	n.d.	n.d.
10	n.d.	3,35 ±0,08 ±0,22	n.d.	3,21 ±0,09 ±0,04	n.d.	n.d.	n.d.	n.d.	n.d.	n.d.	n.d.	n.d.
20	n.d.	4,35 ±0,10 ±0,26	n.d.	4,14 ±0,07 ±0,02	n.d.	3,35 ±0,07 ±0,18	n.d.	n.d.	n.d.	n.d.	n.d.	n.d.
30	n.d.	4,73 ±0,10 ±0,13	3,87 ±0,11 ±0,09	4,22 ±0,07 ±0,11	n.d.	4,42 ±0,11 ±0,30	n.d.	3,56 ±0,10 ±0,24	n.d.	n.d.	n.d.	n.d.
40	n.d.	n.d.	4,12 ±0,08 0,18	n.d.	n.d.	4,49 ±0,10 ±0,30	n.d.	4,29 ±0,07 ±0,07	n.d.	n.d.	n.d.	n.d.
50	3,73 ±0,10 ±0,10	n.d.	4,31* ±0,08 ±0,03	n.d.	n.d.		n.d.	4,33 ±0,07 ±0,11	n.d.	n.d.	n.d.	n.d.
60	4,30 ±0,12 ±0,19	n.d.	n.d.	n.d.	n.d.	n.d.	n.d.	n.d.	n.d.	n.d.	n.d.	n.d.
70	4,30 ±0,09 ±0,12	n.d.	n.d.	n.d.	2,98 ±0,10 ±0,32	n.d.	n.d.	n.d.	n.d.	n.d.	n.d.	n.d.
80	n.d.	n.d.	n.d.	n.d.	3,25 ±0,11 ±0,15	n.d.	n.d.	n.d.	n.d.	n.d.	3,18 ±0,08 ±0,34	n.d.
90	n.d.	n.d.	n.d.	n.d.	3,82 ±0,08 ±0,14	n.d.	n.d.	n.d.	n.d.	n.d.	n.d.	n.d.
100	n.d.	n.d.	n.d.	n.d.	4,27 ±0,08 ±0,07	n.d.	n.d.	n.d.	n.d.	n.d.	n.d.	n.d.
110	n.d.	n.d.	n.d.	n.d.	4,35 ±0,13 ±0,17	n.d.	n.d.	n.d.	n.d.	n.d.	n.d.	3,26 ±0,10 ±0,09

120	n.d.	n.d.	n.d.	n.d.	4,68<br>±0,09<br>±0,14	n.d.	n.d.	n.d.	n.d.	n.d.	n.d.	4,40<br>±0,09<br>±0,23
130	n.d.	n.d.	n.d.	n.d.	n.d.	n.d.	n.d.	n.d.	n.d.	n.d.	n.d.	4,62<br>±0,08<br>±0,06
170	n.d.	n.d.	n.d.	n.d.	n.d.	n.d.	n.d.	n.d.	n.d.	n.d.	3,96<br>±0,07<br>±0,09	n.d.
180	n.d.	n.d.	n.d.	n.d.	n.d.	n.d.	n.d.	n.d.	n.d.	n.d.	4,46<br>±0,10<br>±0,14	n.d.
190	n.d.	n.d.	n.d.	n.d.	n.d.	n.d.	n.d.	n.d.	n.d.	n.d.	4,97<br>±0,09<br>±0,19	n.d.
220	n.d.	n.d.	n.d.	n.d.	n.d.	n.d.	n.d.	n.d.	3,73<br>±0,12<br>±0,07	n.d.	n.d.	n.d.
230	n.d.	n.d.	n.d.	n.d.	n.d.	n.d.	n.d.	n.d.	4,26<br>±0,09<br>±0,28	n.d.	n.d.	n.d.
240	n.d.	n.d.	n.d.	n.d.	n.d.	n.d.	n.d.	n.d.	4,35<br>±0,09<br>±0,04	n.d.	n.d.	n.d.
250	n.d.	n.d.	n.d.	n.d.	n.d.	n.d.	n.d.	n.d.	4,47<br>±0,09<br>±0,09	n.d.	n.d.	n.d.
290	n.d.	n.d.	n.d.	n.d.	n.d.	n.d.	n.d.	n.d.	6,13<br>±0,11<br>±0,09	n.d.	n.d.	n.d.
440	n.d.	n.d.	n.d.	n.d.	n.d.	n.d.	n.d.	n.d.	n.d.	3,73<br>±0,07<br>±0,07	n.d.	n.d.
450	n.d.	n.d.	n.d.	n.d.	n.d.	n.d.	n.d.	n.d.	n.d.	4,29<br>±0,11<br>±0,08	n.d.	n.d.

460	n.d.	n.d.	n.d.	n.d.	n.d.	n.d.	n.d.	n.d.	n.d.	4,44 ±0,12 ±0,12	n.d.	n.d.
550	n.d.	n.d.	n.d.	n.d.	n.d.	n.d.	3,59 ±0,13 ±0,20	n.d.	n.d.	n.d.	n.d.	n.d.
600	n.d.	n.d.	n.d.	n.d.	n.d.	n.d.	4,04 ±0,11 ±0,04	n.d.	n.d.	n.d.	n.d.	n.d.

Table S6: Results of UV C irradiation, 233 nm (LED radiator), stainless steel, n=3, green= inactivation including internal and external consistency ≥ 4 lg, n.d.=not determined

radiation dose [mJ/cm^2]	BCoV	FCoV	SARS CoV-2	VSIV	SuHV-1	MVA	MS 2	PV-1	FCV	MNV	ORV-1	HAdV-5
	lg inactivation (weighting mean ± internal consistency ± external consistency)											
10	0,68<br>±0,10<br>±0,16	2,55<br>±0,10<br>±0,32	1,52<br>±0,09<br>±0,08	1,52<br>±0,11<br>±0,15	0,25<br>±0,11<br>±0,07	0,53<br>±0,09<br>±0,10	0,22<br>±0,10<br>±0,10	2,89<br>±0,08<br>±0,33	1,82<br>±0,10<br>±0,28	0,15<br>±0,11<br>±0,17	0,44<br>±0,10<br>±0,06	0,72<br>±0,10<br>±0,17
50	1,52<br>±0,09<br>±0,06	3,30<br>±0,10<br>±0,22	2,59<br>±0,10<br>±0,08	1,66<br>±0,10<br>±0,06	1,16<br>±0,10<br>±0,18	0,83<br>±0,10<br>±0,10	0,61<br>±0,09<br>±0,10	3,88<br>±0,11<br>±0,06	2,96<br>±0,11<br>±0,04	0,39<br>±0,12<br>±0,15	0,86<br>±0,12<br>±0,07	1,61<br>±0,09<br>±0,10
80	1,56<br>±0,09<br>±0,07	3,32<br>±0,12<br>±0,19	3,48<br>±0,11<br>±0,43	2,83<br>±0,10<br>±0,13	1,32<br>±0,11<br>±0,08	1,61<br>±0,07<br>±0,03	1,50<br>±0,12<br>±0,15	4,28<br>±0,09<br>±0,18	3,28<br>±0,12<br>±0,30	1,73<br>±0,12<br>±0,28	1,49<br>±0,08<br>±0,04	2,88<br>±0,09<br>±0,37

Table S7: Results of UV C irradiation, 233 nm (LED radiator), PVC, n=3, n.d.=not determined

radiationdose [mJ/cm^2]	BCoV	FCoV	SARS CoV-2	VSIV	SuHV-1	MVA	MS 2	PV-1	FCV	MNV	ORV-1	HAdV-5
	lg inactivation (weighting mean ± internal consistency ± external consistency)											
10	0,56<br>±0,11<br>±0,17	0,73<br>±0,09<br>±0,12	0,91<br>±0,10<br>±0,09	0,72<br>±0,10<br>±0,18	0,51<br>±0,09<br>±0,09	0,95<br>±0,11<br>±0,20	0,17<br>±0,12<br>±0,17	1,76<br>±0,09<br>±0,18	1,56<br>±0,09<br>±0,23	0,82<br>±0,11<br>±0,15	0,74<br>±0,08<br>±0,09	1,61<br>±0,11<br>±0,05
50	1,10<br>±0,11<br>±0,23	1,41<br>±0,11<br>±0,17	1,78<br>±0,12<br>±0,15	1,55<br>±0,13<br>±0,07	1,29<br>±0,11<br>±0,31	1,86<br>±0,11<br>±0,18	0,44<br>±0,12<br>±0,18	1,84<br>±0,10<br>±0,24	2,03<br>±0,10<br>±0,12	0,92<br>±0,09<br>±0,09	1,48<br>±0,10<br>±0,14	1,87<br>±0,11<br>±0,06
80	1,40<br>±0,10<br>±0,06	2,77<br>±0,11<br>±0,30	2,88<br>±0,13<br>±0,18	2,00<br>±0,10<br>±0,16	1,73<br>±0,09<br>±0,11	2,76<br>±0,08<br>±0,09	0,44<br>±0,09<br>±0,25	2,48<br>±0,17<br>±0,17	2,51<br>±0,15<br>±0,06	1,46<br>±0,11<br>±0,07	1,79<br>±0,08<br>±0,06	2,67<br>±0,11<br>±0,17

Table S8: Results of UV C irradiation, 233 nm (LED radiator), glass, n=3, n.d.=not determined

radiationdose [mJ/cm^2]	BCoV	FCoV	SARS CoV-2	VSIV	SuHV-1	MVA	MS 2	PV-1	FCV	MNV	ORV-1	HAdV-5
	lg inactivation (weighting mean ± internal consistency ± external consistency)											
10	0,46<br>±0,10<br>±0,08	1,72<br>±0,09<br>±0,14	1,74<br>±0,09<br>±0,16	1,84<br>±0,09<br>±0,14	0,85<br>±0,08<br>±0,09	0,94<br>±0,10<br>±0,16	0,88<br>±0,11<br>±0,13	0,72<br>±0,10<br>±0,12	1,48<br>±0,10<br>±0,14	0,36<br>±0,06<br>±0,10	0,86<br>±0,09<br>±0,11	1,80<br>±0,12<br>±0,15

50	0,65<br>±0,10<br>±0,15	2,15<br>±0,11<br>±0,20	2,44<br>±0,11<br>±0,09	2,17<br>±0,10<br>±0,17	1,76<br>±0,08<br>±0,08	1,52<br>±0,12<br>±0,16	1,03<br>±0,11<br>±0,24	2,12<br>±0,12<br>±0,17	2,68<br>±0,11<br>±0,15	1,05<br>±0,13<br>±0,10	1,11<br>±0,11<br>±0,24	2,24<br>±0,12<br>±0,20
80	1,67<br>±0,10<br>±0,26	2,79<br>±0,11<br>±0,33	2,96<br>±0,10<br>±0,26	2,61<br>±0,11<br>±0,32	2,22<br>±0,10<br>±0,32	2,18<br>±0,08<br>±0,28	1,34<br>±0,11<br>±0,29	2,85<br>±0,11<br>±0,11	3,18<br>±0,11<br>±0,19	1,59<br>±0,08<br>±0,06	1,84<br>±0,12<br>±0,13	2,92<br>±0,08<br>±0,14

Figure S1: Absorption of radiation by cell culture medium without and with FCS as well as natural and artificial saliva that was used in former studies.

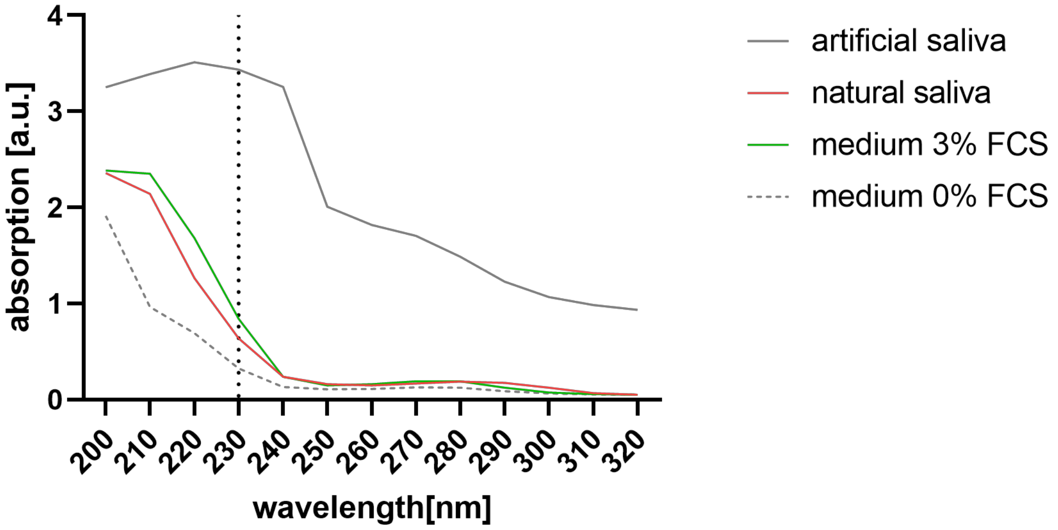

Supplement: Supplementary file 1 [file viruses-16-01904-s001.zip › viruses-3285119-supplementary.pdf]
